# A High-Content Screen for the Identification of Plant Extracts with Insulin Secretion-Modulating Activity

**DOI:** 10.3390/ph14080809

**Published:** 2021-08-17

**Authors:** Roland Hager, Johannes Pitsch, Jakob Kerbl-Knapp, Cathrina Neuhauser, Nicole Ollinger, Marcus Iken, Josef Ranner, Verena Mittermeier-Kleßinger, Corinna Dawid, Peter Lanzerstorfer, Julian Weghuber

**Affiliations:** 1 School of Engineering, University of Applied Sciences Upper Austria, 4600 Wels, Austria; roland.hager@fh-wels.at (R.H.); johannes.pitsch@fh-wels.at (J.P.); jakob.kerbl-knapp@medunigraz.at (J.K.-K.); cathrina.neuhauser@fh-wels.at (C.N.); 2 FFoQSI—Austrian Competence Center for Feed and Food Quality, 3430 Tulln, Austria; nicole.ollinger@ffoqsi.at; 3 PM International AG, 5445 Schengen, Luxembourg; marcus.iken@pm-international.com; 4 Food Chemistry and Molecular Sensory Science, Technical University of Munich, 85354 Freising, Germany; josef.ranner@tum.de (J.R.); verena.mittermeier@tum.de (V.M.-K.); corinna.dawid@tum.de (C.D.)

**Keywords:** insulin, luciferase, natural plant extracts, bioactives, diabetes, β cells, screening, GC-MS, LC-MS, Western blotting, natural compounds

## Abstract

Bioactive plant compounds and extracts are of special interest for the development of pharmaceuticals. Here, we describe the screening of more than 1100 aqueous plant extracts and synthetic reference compounds for their ability to stimulate or inhibit insulin secretion. To quantify insulin secretion in living MIN6 β cells, an insulin–*Gaussia* luciferase (Ins-GLuc) biosensor was used. Positive hits included extracts from *Quillaja saponaria*, *Anagallis arvensis*, *Sapindus mukorossi*, *Gleditsia sinensis* and *Albizia julibrissin*, which were identified as insulin secretion stimulators, whereas extracts of *Acacia catechu*, *Myrtus communis*, *Actaea spicata* L., *Vaccinium vitis-idaea* and *Calendula officinalis* were found to exhibit insulin secretion inhibitory properties. Gas chromatography-mass spectrometry (GC-MS) and liquid chromatography-mass spectrometry (LC-MS) were used to characterize several bioactive compounds in the selected plant extracts, and these bioactives were retested for their insulin-modulating properties. Overall, we identified several plant extracts and some of their bioactive compounds that may be used to manipulate pancreatic insulin secretion.

## 1. Introduction

**Figure 1:**
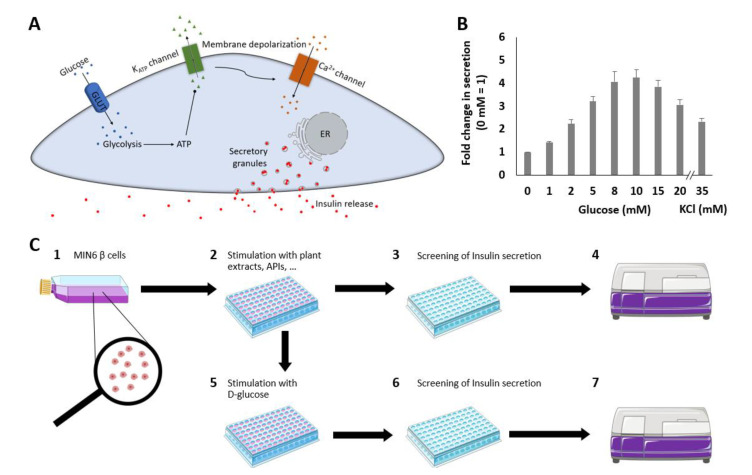
Schematic overview of the GSIS pathway from pancreatic β cells that produce and secrete insulin in response to changes in ambient blood glucose concentrations. Glucose enters the cell via the glucose transporter GLUT2 and is metabolized to pyruvate and ATP. The generated ATP binds to and closes ATP-dependent potassium channels (K_ATP_ channels). Due to channel closure, potassium exit is blocked, resulting in depolarization of the cell membrane. Voltage-gated calcium channels are thus triggered, and an influx of calcium occurs. The elevated cytoplasmic calcium concentration triggers the release of insulin and C-peptide in equimolar amounts (**A**). Insulin secretion depending on different glucose concentrations in MIN6 β cells and in response to 35 mM KCl. Fold changes in the secreted luciferase activity expressing Ins-GLuc normalized to the activity of 0 mM glucose and expressed as fold change ± SEM. Data are the average of at least three independent experiments with a minimum of 17 replicates in total (**B**). Schematic overview of the insulin secretion stimulation and suppression assay (**C**). MIN6 β cells were cultured in flasks or dishes, trypsinized, counted and diluted in cell culture media (1). Cells (200 µL) were aliquoted into wells of a 96-well plate and cultured before washing and starving in KRPH buffer and incubation with plant extracts (2). Fifty microliters of supernatant were removed, pipetted into a white 96-well plate and mixed with working solution (3). Luminescence was measured immediately after pipetting (4). To test the suppression of insulin secretion of the plant extracts, 10 mM glucose was added (5) after incubation with different plant extracts (2). Assay preparation and measurements (6, 7) were performed as described previously (3, 4).

Metabolic diseases are global health problems that are rapidly increasing worldwide. In this regard, energy metabolism represents a key player that is controlled by insulin secretion from pancreatic β cells. Glucose-stimulated insulin secretion (GSIS) in these cells is controlled by various factors [1]. When the ambient blood glucose concentration increases, glucose is transported by selective transporters into β cells. Elevated glucose levels induce intracellular energy and metabolic processes with a subsequent increase in the ATP/ADP ratio followed by the closure of ATP-triggered potassium (K_ATP_) channels. Due to the inhibition of K_ATP_ channels, the exit of potassium from cells is blocked, resulting in membrane depolarization. Voltage-dependent Ca^2+^ channels (VDCCs) are thus activated, allowing Ca^2+^ influx; this increase in cytosolic Ca^2+^ concentration then initiates GSIS. This triggering pathway is followed by a time-dependent increase in insulin secretion [2,3]. A proposed simplified network of insulin exocytosis from pancreatic β cells is shown in Figure 1A. The intracellular network for the regulation of GSIS is very complex and multifactorial. A large number of factors, including mediators of the autonomous nervous system, hormones and nutrients, must be considered [4,5].

Currently, there are numerous antidiabetic agents available for the treatment of diabetes mellitus (DM), which target different receptors [6]. The most important classes of antidiabetic oral medicines include biguanides, such as metformin, sulfonylureas, meglitinide, thiazolidinedione, dipeptidyl peptidase 4 inhibitors, sodium glucose cotransporter (SGLT2) inhibitors and α-glucosidase inhibitors [7,8]. Sulfonylureas increase insulin secretion by blocking K_ATP_ channels and therefore lower blood glucose levels. They are divided into first-generation agents, such as tolbutamide, chlorpropamide, acetohexamide, metahexamide and tolazamide, and second-generation agents, such as glipizide, glyburide, gliclazide, glibenclamide and glimepiride, which are sometimes also considered third-generation agents [9]. Another class of drugs for the treatment of DM is meglitinides (glinides), which include repaglinide and nateglinide. Diazoxide (DZ) is a direct insulin secretion inhibitor that is often used for the treatment of insulinoma, a rare neuroendocrine tumor of the pancreas that leads to hypoglycemia. DZ inhibits insulin release by opening K_ATP_ channels, in contrast to sulfonylureas, which stimulate insulin secretion by blocking K_ATP_ channels [10].

Medicinal plants are used extensively as drugs for various diseases. Especially in developing countries, medicinal plants are used to treat DM due to the costs of conservative medicines [11]. Medicinal plants are a source of biological and chemical compounds that are important pharmaceuticals and are currently an important tool for the identification of novel drug lead compounds. A large number of plants, their extracts and their phytochemicals have been shown to affect the insulin secretion mechanism [12]. *Galega officinalis*, a plant that contains biguanide, has been used since the middle ages for the treatment of diabetes [13]. Several plant species are known for their antidiabetic properties, and a variety of plant extracts have been described to have valuable antidiabetic treatment effects. Importantly, these plants and their extracts are considered to be less toxic and have fewer side effects than synthetic drugs [14,15,16]. On the one hand, plant extracts can be used as complementary and alternative remedies to prevent metabolic diseases, and on the other hand, they are an interesting source of compounds for potential new drug candidates [17].

Modern screening techniques allow for the discovery of new bioactive compounds from plant extracts and other biological sources. Chemical screening techniques such as liquid chromatography/nuclear magnetic resonance (LC/NMR), LC-MS or liquid chromatography/ultraviolet (LC/UV) detection provide structural information that can lead to the identification of novel compounds, and the targeted isolation of constituents presenting unknown spectroscopic features can be performed [18]. In addition to chemical screening, bioassays that are adaptable to the testing of plant extracts must be undertaken. High-throughput screening (HTS) platforms for different types of cells and cell culture systems have been developed and are used for drug discovery to test a large number of compounds within a short time period. These systems have led to many drug discoveries using natural products [16,17,19].

For medications such as sulfonylureas and DZ, their functional modes of action have been well analyzed. However, for plant extracts and the natural compounds they contain, such information is mostly lacking, mainly due to their synergistic effects. Herein, we report several different plant extracts and some of their bioactive compounds that have been identified by a high-content screen to modulate insulin secretion in living β cells.

## 2. Results and Discussion

### 2.1. GSIS from MIN6 β Cells

Mouse MIN6 β cells were used based on an assay described by Kalwat et al. [20], which was adapted for our high-content screening approach. Insulin secretion, which depends on the extracellular glucose concentration, was measured to validate the sensor system (Figure 1B). Stimulation by increasing glucose concentrations resulted in elevated insulin secretion rates, with a maximum response at 10 mM glucose (4.2-fold). Using higher concentrations of glucose led to a decrease in insulin secretion, which is in line with other studies [21,22]. Therefore, we chose 10 mM glucose for subsequent screening experiments.

KCl can also be used to mimic depolarization during GSIS and is considered equivalent to the first phase of GSIS [23]. KCl in combination with DZ and glucose has been shown to induce insulin secretion [20]. Accordingly, the stimulation of MIN6 cells by KCl also affected the insulin secretion rate (2.3-fold).

### 2.2. Modulation of Insulin Secretion with Different Kinds of Pharmaceuticals

**Figure 2:**
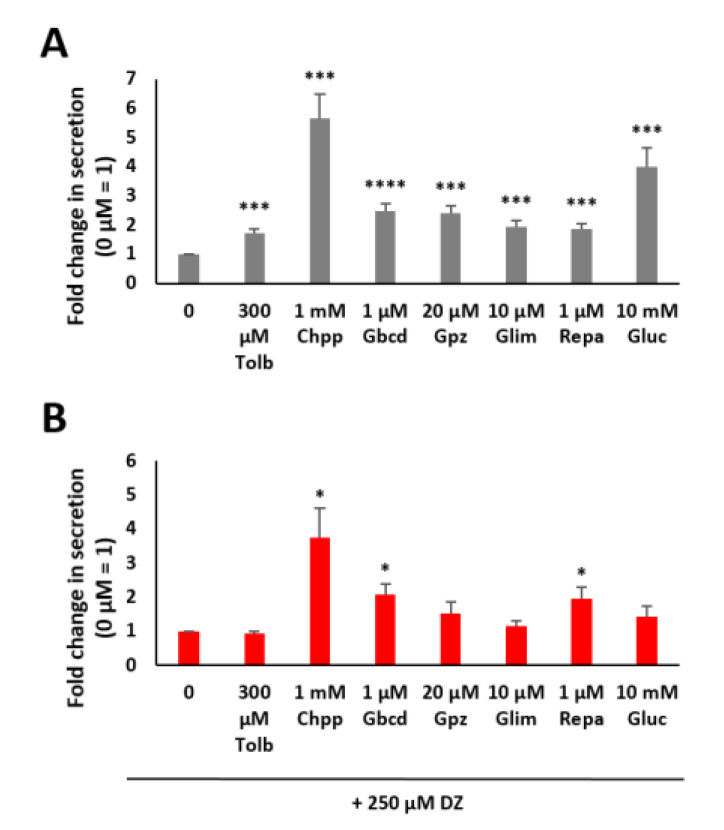
Fold change in insulin secretion as measured by luciferase activity in MIN6 β cells after incubation with tolbutamide (Tolb), chlorpropamide (Chpp), glibenclamide (Gbcd), glipizide (Gpz), glimepiride (Glim), repaglinide (Repa) and glucose (Gluc) (**A**) and incubation after preincubation with 250 µM diazoxide (DZ) (**B**). Fold change in insulin secretion as measured by luciferase activity in MIN6 β cells expressing Ins-GLuc normalized to the activity of 0 mM glucose and expressed as the means ± SEM (*n* ≥ 8). * *p* < 0.05; *** *p* < 0.001; **** *p* < 0.0001.

**Figure A1:**
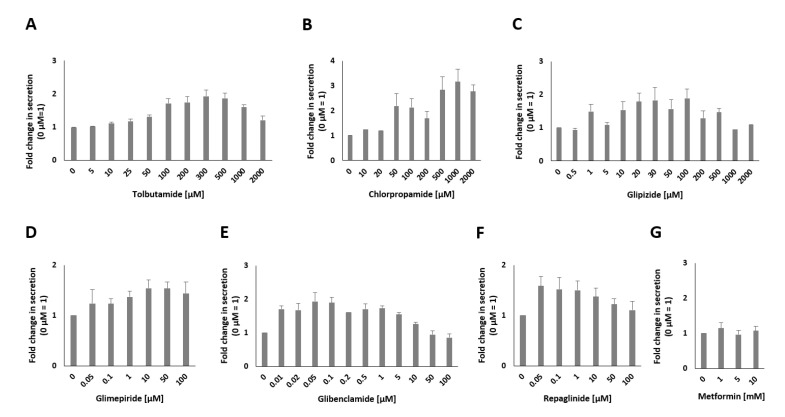
Fold change in the amount of insulin secreted expressed as luciferase activity in MIN6 β cells after incubation with different concentrations of tolbutamide (**A**), chlorpropamide (**B**), glipizide (**C**), glimepiride (**D**), glibenclamide (**E**), repaglinide (**F**) and metformin (**G**).

**Figure A2:**
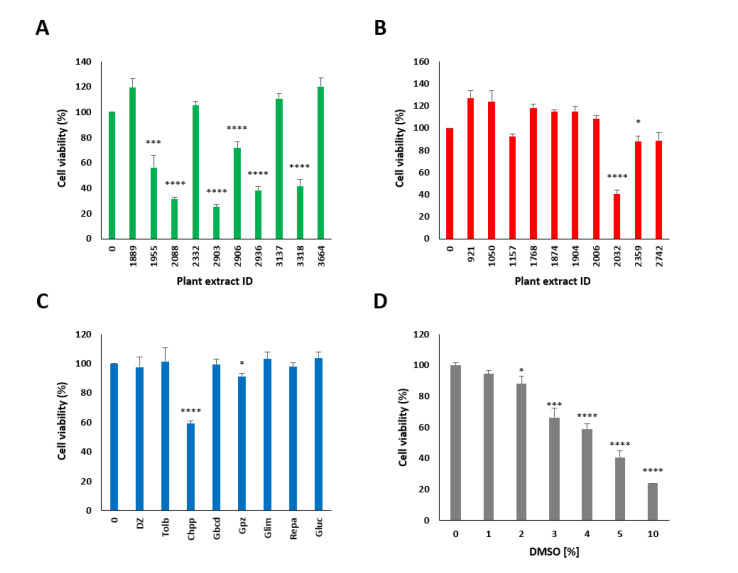
Cell viability of MIN6 pancreatic β cells determined by the resazurin assay tested at a concentration of 10 µg/mL. Stimulating plant extracts: 1889: *Populus nigra*, 1955: *Quillaja saponaria*, 2088: *Anagallis arvensis*, 2332: *Allium sativum*, 2903: *Sapindus mukorossi*, 2906: *Sapindus mukorossi*, 3137: *Albizia julibrissin*, 2936: *Quillaja saponaria*, 3318: *Gleditsia sinensis* and 3664: *Lycium barbarum*, (**A**). Suppressing plant extracts: 921: *Calendula officinalis*, 1050: *Rosae*, 1157: *Vaccinium vitis-idaea*, 1768: *Cistus incanus*, 1874: *Vaccinium vitis-idaea*, 1904: *Myrtus communis*, 2006: *Actaea spicata* L., 2332: *Acacia catechu*, 2359: *Filipendula ulmaria* and 2742: *Terminalia arjuna* (**B**). Active pharmaceutical ingredients: 250 µM DZ, 300 µM tolbutamide, 1 mM chlorpropamide, 1 µM glibenclamide, 20 µM glipizide, 10 µM glimepiride and 1 µM repaglinide (**C**). DMSO (**D**). Data are normalized to untreated cells (0 mM glucose or 0% DMSO = 100% viability) and presented as the mean ± SEM. *n* ≥ 8; **** p < 0.0001, *** p < 0.001, and * p < 0.05 indicate a significant difference compared to the untreated cells.

Different types of sulfonylureas are widely used to treat type 2 diabetes mellitus (T2DM) [24,25]. Hence, we tested the effects of tolbutamide, chlorpropamide, glipizide, glibenclamide and glimepiride, all of which are sulfonylureas, for their abilities to stimulate insulin secretion. Additionally, repaglinide, a nonsulfonylurea insulin secretagogue that belongs to the class of meglitinides [26], was tested under different conditions, as shown in Figure 2. A dose–response relationship of the different pharmaceuticals was also determined to estimate the concentration that was best suited for our experiments, as outlined in Figure A1. The range in the concentration of the different pharmaceuticals was defined as suggested in the literature [27,28,29]. The effects of diverse insulinotropic compounds on the viability of MIN6 β cells are shown in Figure A2C. The use of chlorpropamide at the indicated concentration resulted in a significant decrease in cell viability to 59%. Glipizide also showed a decrease in viability (91%). Therefore, the interpretation of the data for these two insulinotropic compounds is challenging.

We analyzed the abovementioned six insulinotropic compounds and found that all significantly increased insulin secretion in MIN6 β cells (Figure 2A). Chlorpropamide at the chosen concentration of 1 mM led to the highest increase; a 5.7-fold increase in insulin secretion was observed compared to a 4-fold increase when using glucose.

The effectiveness of all of the other drugs was lower than that of glucose but still significantly increased insulin secretion (tolbutamide 1.7-fold, glibenclamide 2.5-fold, glipizide 2.4-fold, glimepiride 1.9-fold and repaglinide 1.9-fold). A comparable study described that secretagogues such as KCl or glipizide trigger insulin secretion in the absence of glucose, but glucose amplifies the amount secreted in a dose-dependent manner. Other insulinotropic substances increase the amount of secreted insulin mainly in the presence of permissive glucose levels [29].

Furthermore, we analyzed insulin secretion in MIN6 β cells that were preincubated with 250 µM DZ for 1 h. The K_ATP_ channels were opened by DZ, and stimulation of insulin secretion by glucose was not possible because the β cell membrane was no longer depolarized [3,5]. As expected, 10 mM glucose did not lead to a significant effect under these experimental conditions (Figure 2B). In addition, there was also no significant difference in insulin secretion after stimulation with tolbutamide, glipizide or glimepiride. However, chlorpropamide (3.7-fold), glibenclamide (2.1-fold) and repaglinide (2-fold) consistently increased insulin secretion even after preincubation with DZ.

Additionally, the effect of metformin was tested for its influence on the modulation of insulin secretion (see Figure A1G). Metformin, a biguanide antihyperglycemic agent, is known to act mainly by increasing the action of insulin in muscle and liver tissue; thus, it is widely used in the treatment of T2DM [30]. In our tests, metformin did not show any significant effects. This result is in line with the available literature, describing that metformin does not play an important role in either promoting or inhibiting insulin secretion [31].

In conclusion, our experimental setup allows for the specific identification of compounds with insulin secretion-modulating properties.

### 2.3. Screening and Characterization of Insulin Secretion-Modulating Plant Extracts

**Figure 3:**
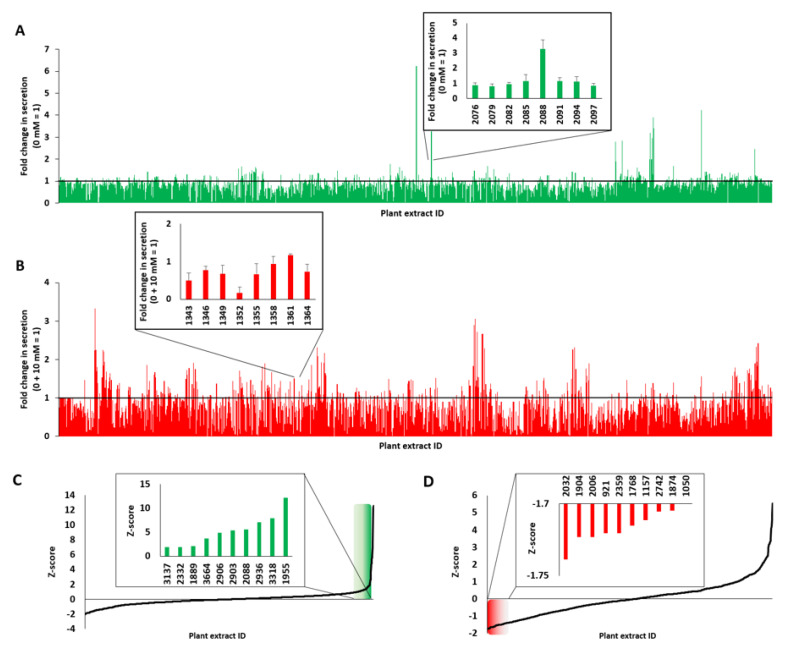
Ins-Gluc-expressing MIN6 β cells were treated with more than 1100 plant extracts (**A**), green illustrated data or preincubated with these extracts and diazoxide (DZ) for 1 h and stimulated with 10 mM glucose (**B**), red illustrated data. The Z-score was calculated from normalized values, and the data were sorted and are illustrated in (**C**) for incubation with plant extracts and (**D**) for incubation with plant extracts in combination with 10 mM glucose. Plant extracts were screened at a final concentration of 10 µg/mL (*n* = 4).

To identify plant extracts with potential antidiabetic activity, more than 1100 samples were screened. All aqueous plant extracts were provided by the open-access screening library plant extract collection Kiel in Schleswig-Holstein (PECKISH), which was fabricated as described by Onur et al. [32]. It has been shown that comparable screening systems can be automated and adapted to 384-well or 1536-well formats [20,29]. Figure 3 represents a summary and ranking of all of the screened plant extracts in our study. The obtained data for the plant extracts that stimulated insulin secretion are indicated in Figure 3A, and those that inhibited insulin secretion are shown in Figure 3B. The small sections display an enlarged view of the particular extracts. Normalization of the data from the stimulation screen to the activity of 0 mM glucose resulted in a list of 316 plant extracts that showed higher values than untreated cells. The results ranked by z-score are presented in Figure 3C, and the ten extracts showing the highest stimulation property values (see inlet) were analyzed in further detail. Regarding the insulin secretion suppressing properties, we normalized the data to the activity of 0 + 10 mM glucose and obtained a list of 831 plant extracts that gave values less than 1. The data from the insulin secretion suppressing plant extracts ranked by z-score are shown in Figure 3D, and the plant extracts showing the lowest values were analyzed in further detail.

We found that the extracts of black poplar (*Populus nigra*), scarlet pimpernel (*Anagallis arvensis*), garlic (*Allium sativum*), pink silk tree (*Albizia julibrissin*), Chinese honey locust (*Gleditsia sinensis*), boxthorn (*Lycium barbarum*), two different extracts of the soap bark tree (*Quillaja saponaria*) and two different extracts of reetha (*Sapindus mukorossi*) were among the ten highest plant extracts with stimulatory properties.

We also identified the ten best plant extracts that inhibited insulin secretion: common marigold (*Calendula officinalis*), rose (*Rosae*), cistus (*Cistus incanus*), common myrtle (*Myrtus communis*), herb christopher (*Actaea spicata* L.), black cutch (*Acacia catechu*), meadowsweet (*Filipendula ulmaria*), arjun tree (*Terminalia arjuna*) and two different extracts of lingonberry (*Vaccinium vitis-idaea*). The effects of DZ were examined and compared to the results of the plant extracts that suppressed insulin secretion. In our in vitro screening assay, 201 aqueous plant extracts showed higher efficacy than DZ, which is known to inhibit insulin secretion by opening K_ATP_ channels. Therefore, DZ is used for the treatment of hypoglycemia caused by conditions that cause the pancreas to release too much insulin, such as insulinomas [21]. Its underlying mechanism of action is not fully understood because of the high complexity of the composition of most plant extracts and the fact that a certain ratio of many different compounds may play an important role.

**Figure 4:**
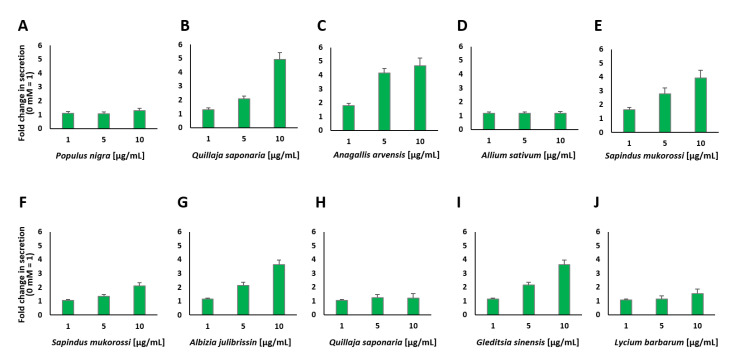
Insulin secretion from MIN6 β cells in response to stimulation with various concentrations of the indicated plant extracts (**A**–**J**). Fold change in the amount of secreted insulin expressed as luciferase activity from Ins-GLuc normalized to the activity of 0 mM glucose and expressed as fold change ± SEM (*n* ≥ 8).

**Figure A3:**
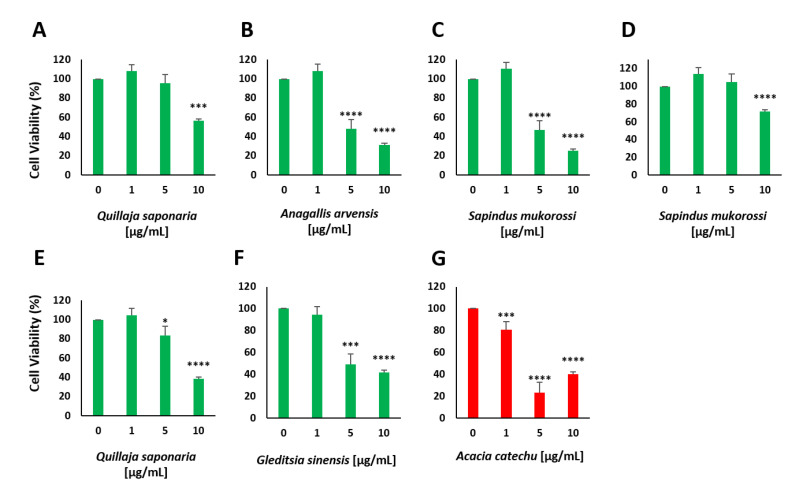
Cell viability of MIN6 pancreatic β cells after treatment with different concentrations of different extracts as determined by the resazurin assay. *Quillaja saponaria* (**A**), *Anagallis arvensis* (**B**), *Sapindus mukorossi* (**C**), *Sapindus mukorossi* (**D**), *Quillaja saponaria* (**E**), *Gleditsia sinensis* (**F**) and *Acacia catechu* (**G**). * *p* < 0.05; *** *p* < 0.001; **** *p* < 0.0001.

The plant extracts that showed the highest values for insulin secretion stimulation were tested again at different concentrations, as indicated in Figure 4. We chose concentrations from 1 to 10 µg/mL, as it was found that some of the plant extracts showed toxic effects at 10 µg/mL (see Figure A2). At 1 µg/mL, none of the plant extracts displayed a significant decrease in cell viability, while 5 µg/mL led to a negative influence on cell viability for the four extracts (see Figure A3A–F).

Interestingly, two different aqueous plant extracts prepared from *Q. saponaria* revealed divergent results (see Figure 4B,H). This plant has already been described to repress hyperglycemia after combining it with *Yucca schidigera* into the diet of diabetic animals [33]. One of the *Q. saponaria* extracts showed a very high increase in insulin secretion (5.2-fold) at a concentration of 10 µg/mL, but this concentration also resulted in a significant decrease in cell viability (56%, see Figure A3A). The concentration of 5 µg/mL also resulted in an increase in insulin secretion (2.2-fold). In contrast, the second *Q. saponaria* extract did not show such a strong increase, which indicates that the preparation of the extracts and the parts of the plant used to play an important role in the impact of the aqueous extract and the metabolic reaction of the treated cells. Similar effects caused by different parts of the same plant, e.g., the berries and roots, are known to occur with other extracts, such as ginseng [34]. The second extract of *Q. saponaria* showed just a 1.3-fold increase in insulin secretion at the highest concentration used and a decrease in cell viability to ~40%. The collection and handling of plant material, as well as the fabrication and extraction procedures of the different plant extracts, are described elsewhere [32]. *A. arvensis*, *S. mukorossi* and *G. sinensis* also showed a significant decrease in cell viability when used at a concentration of 10 µg/mL (see Figure A2 and Figure A3). *A. arvensis*, which was identified as a potential rich source of compounds with antidiabetic activity [35], led to an increase in insulin secretion when applied at a concentration of 5 µg/mL. *P. nigra* and *A. sativum* demonstrated minor effects at all chosen concentrations, but similar to *A. julibrissin* and *L. barbarum*, they did not show a significant decrease in cell viability. Both *P. nigra* and *A. sativum* have been described in the literature to have antidiabetic potential, and it has also been demonstrated that poplar buds can regulate the blood glucose levels of diabetic mice and ameliorate the abnormalities in glycometabolism, dyslipidemia and inflammation caused by T2DM [36,37]. The purified components of *L. barbarum* were therefore characterized as useful adjuvants for the treatment of diabetes and its related illnesses [38]. *A. Julibrissin* showed a 3.8-fold increase in insulin secretion at a concentration of 10 µg/mL and a 2.2-fold increase at a concentration of 5 µg/mL. With our screening method, we found several plant extracts that increased insulin secretion, and some of these extracts also influenced cell viability at a certain concentration, as presented in Figure A2 and Figure A3.

**Figure 5:**
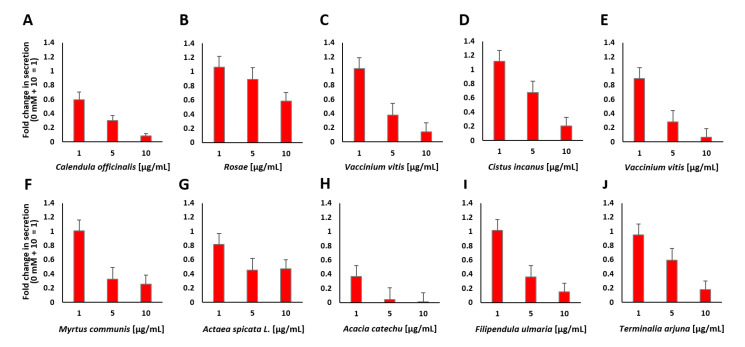
Insulin secretion from MIN6 β cells in response to stimulation with 10 mM glucose after preincubation with various concentrations of the indicated plant extracts (**A**–**J**). Fold change in the amount of secreted insulin expressed as luciferase activity from Ins-GLuc normalized to the activity of 0 mM + 10 mM glucose expressed as fold change ± SEM (*n* ≥ 11).

The plant extracts that showed the lowest values for insulin secretion after preincubation and stimulation with 10 mM glucose were also tested at different concentrations, as outlined in Figure 5.

Only *A. catechu*, as a representative plant extract that showed insulin secretion inhibitory properties, was associated with a significant decrease in cell viability (40%) at the tested concentrations (see Figure A2B and Figure A3G). However, the *A. catechu* extract failed to inhibit insulin secretion after preincubation at 1 µg/mL followed by treatment with 10 mM glucose. All other tested plant extracts did not affect cell viability, but they significantly decreased insulin secretion. According to the z-score rankings, *M. communis*, *A. spicata* and *C. officinalis* are associated with strong inhibition of insulin secretion. *C. officinalis* displayed 0.1-fold insulin secretion at a concentration of 10 µg/mL, a very low number compared to that of DZ, which showed a 0.4-fold value of insulin secretion at a concentration of 250 µM. *C. officinalis* at concentrations of 1 and 5 µg/mL also showed a strong decrease in insulin secretion (0.5-fold and 0.3-fold, respectively). Two different plant extracts of *V. vitis-idaea* were analyzed, and both showed a decrease at a concentration of 10 µg/mL to 0.14-fold and 0.07-fold, respectively. It has also been reported that lingonberry (*V. vitis-idaea*) extracts are used for the treatment of T2DM [39]. Several plants with hypoglycemic properties, such as ginseng or bitter melon, have already been described. It has also been shown that different parts of these plants show different effects. Ginseng berries seem to have more potent antihyperglycemic activity than ginseng roots [34]. Many in vitro and in vivo studies have demonstrated the beneficial effects of plant extracts or phytochemicals for the treatment of diabetes [40].

Advantages of the current assay used are its low cost and the minimal time expenditure of the luciferase-based screening method compared to other approaches, such as enzyme-linked immunosorbent assays (ELISAs). It has been shown that direct measurement of insulin secretion via ELISA highly correlates with GLuc secretion [41]. A limitation of the system is that only secretion, not the expression of insulin, can be detected.

### 2.4. Chemical Analysis of Plant Extracts and the Influence of the Identified Bioactive Compounds on Insulin Release

To identify the putative bioactive compounds in the selected plant extracts, we performed GC-MS and LC-MS. Therefore, four plant extracts with insulin secretion stimulatory properties (*A. arvensis*, *A. sativum*, *G. sinensis* and *L. barbarum*) and four with inhibitory properties (*C. officinalis*, *V. vitis-idaea*, *Actaea spicata L.* and *F*. *ulmaria*) were chosen for the characterization. For GC-MS analysis we derivatized above mentioned plant extracts with N,O-bis(trimethylsilyl)trifluoroacetamide (BSTFA). The results of GC-MS analysis are depicted in Figure A5 and Figure A6. The results of the LC-MS analysis are shown in Figure A7. Plant extracts were chosen based on their strong modulating effects on insulin secretion or because of their availability in central Europe.

As a result of the GC-MS characterization, quercetin and myricetin were detected in all analyzed samples at similar concentrations, regardless of whether the plant extracts were identified as stimulators or inhibitors of insulin secretion. Therefore, the different effects based solely on these two compounds cannot be explained. *L. barbarum* contained 2.4-fold more quercetin than *G. sinensis*, and the amount of myricetin in *L. barbarum* was 2.7-fold higher. It has already been shown that the flavonoid quercetin and its glycoside rutin can stimulate insulin release in INS-1 cells [42]. The beneficial antidiabetic properties of myricetin were also outlined using cultured cells and diabetic animals [43]. Scopoletin, although at low levels, was detected in only the *C. officinalis* extract. Coumarins such as scopoletin reduce blood glucose levels and improve insulin sensitivity. Treatment with scopoletin has been shown to increase glucose uptake in 3T3-L1 adipocytes in a dose-dependent manner [44]. Caffeic acid was present in all analyzed samples but at a much higher level in only the *F. ulmaria* extract, which contained ~40 times more caffeic acid than the *L. barbarum* extract. Catechin was found to be the most abundant compound in the *V. vitis-idaea* extract. It contained ~50 times more catechin than *A. spicata*. Furthermore, catechin was found in small amounts in *A. arvensis* and *F. ulmaria.* The flavonoid catechin is known to be a powerful antioxidant and anti-inflammatory molecule that is found in a variety of plants. A combination of catechin, epicatechin and rutin was successfully tested in alloxan-induced diabetic mice as an antidiabetic drug alternative [45]. Polyphenolic acid chlorogenic acid has been described as having beneficial metabolic effects on glucose homeostasis. The highest concentration of chlorogenic acid was found in *F. ulmaria*, which contained ~15 times more of this compound than that found in *L. barbarum.* In addition, chlorogenic acid was also found in small amounts in *C. officinalis*, *V. vitis-idaea* and *A. arvensis.* Chlorogenic acid isolated from *Cecropia obtusifolia* possesses a broad range of pharmacological properties, such as anticarcinogenic, neuroprotective, antioxidant, anti-inflammatory, hypoglycemic and hypolipidemic properties [46,47]. *C. obtusifolia* has also been described as an insulin secretion-increasing agent in RINm5F cells, and it increases the mRNA expression of PPARγ and GLUT4 [48]. Generally, little is known about the underlying mechanisms of how certain plant extracts and their bioactive compounds regulate and influence intracellular pathways.

Furthermore, we used LC-MS as an additional method for the identification of supposed bioactive compounds in the selected plant extracts. To identify possible saponins, an in silico database with structural information of various saponins was used [49,50,51,52]. We compared the list of compounds with this database and filtered them for possible hits, as shown in Figure A7. Tentative hits for the *C. officinalis* extract were oleanolic acid diglucoside, oleanolic acid monoglucoside, oleanolic acid monoglucuronide, oleanolic acid monoglucuronide diglucoside, oleanolic acid monoglucuronide monoglucoside, oleanolic acid tetraglucoside, oleanolic acid triglucoside and parillin. In *A. sativum*, gitogenin 3-O-tetrasaccharide and voghieroside E1/E2 were found to be tentative compounds. For *A. arvensis*, LC-MS analysis resulted in several potential hits, including anagallisin A, anagallisin B, anagallisin C, oleanolic acid tetraglucoside, oleanolic acid triglucoside and parillin, and in *G. sinensis*, we found 5,6-dihydrosolanine, anagallisin C, gleditsia saponin E’, gleditsioside H, gleditsioside I, gleditsioside J, gleditsioside K, oleanolic acid diglucoside, oleanolic acid triglucoside and voghieroside D1/D2. For *V. vitis-idaea*, only gracillin was identified as a possible hit. LC-MS analysis was not successful in identifying potential bioactive compounds in *L. barbarum*, *A. spicata* and *F. ulmaria.*

**Figure 6:**
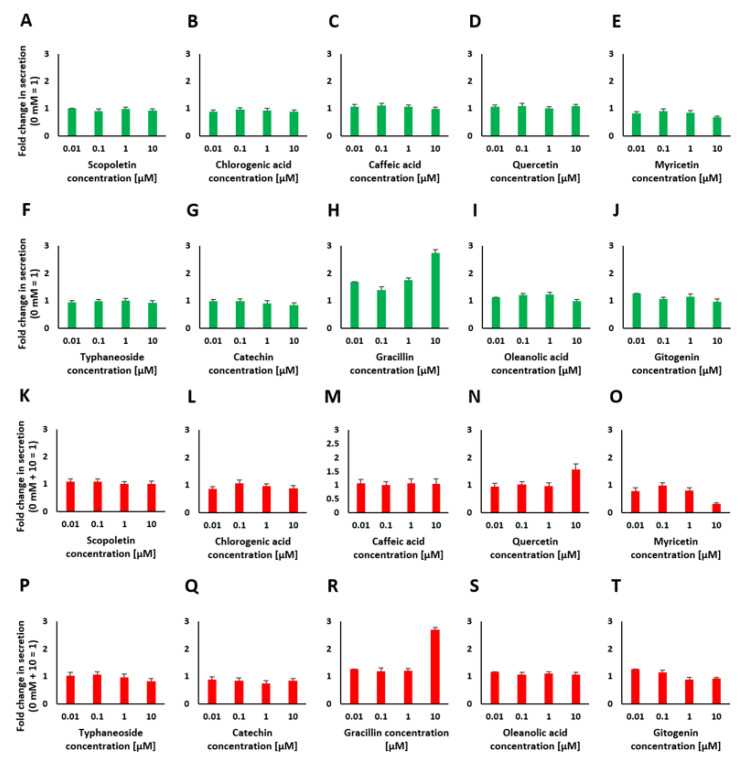
Insulin secretion in response to stimulation with various concentrations of identified bioactives (**A**–**J**) and insulin secretion in response to stimulation with 10 mM glucose after preincubation with various concentrations of bioactives to test for insulin secretion inhibiting properties (**K**–**T**).

**Figure A4:**
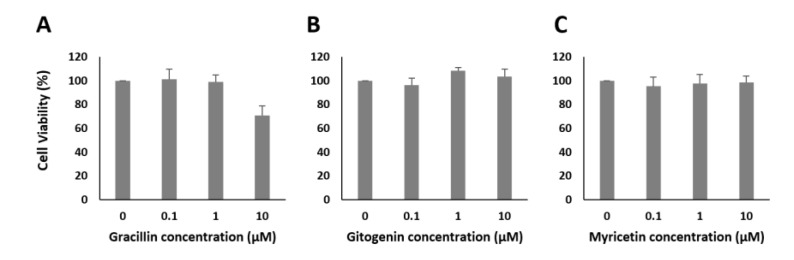
Cell viability of MIN6 pancreatic beta cells dependent on different concentrations of selected bioactive compounds: (**A**) Gracillin, (**B**) Gitogenin and (**C**) Myricetin.

**Figure A5:**
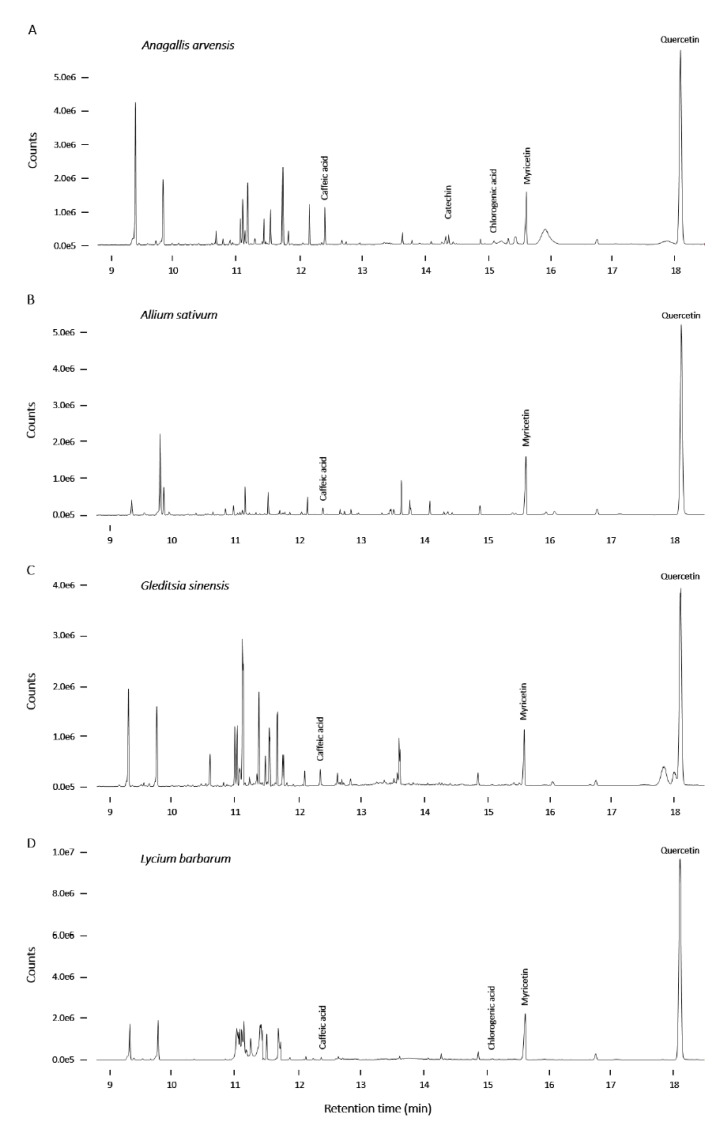
GC-MS chromatograms of Anagallis arvensis (**A**), Allium sativum (**B**), Gleditsia sinensis (**C**) and Lycium barbarum (**D**).

**Figure A6:**
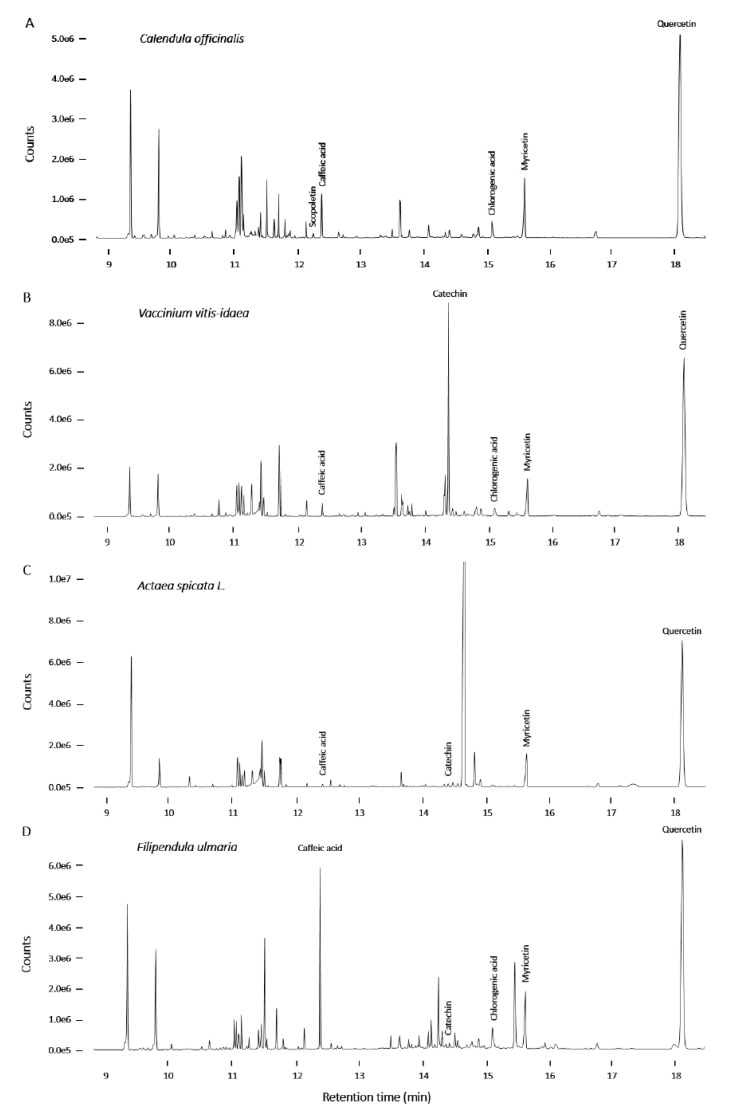
GC-MS chromatograms of *Calendula officinalis* (**A**), *Vaccinium vitis-idaea* (**B**), *Actaea spicata* L. (**C**) and *Filipendula ulmaria* (**D**).

**Figure A7:**
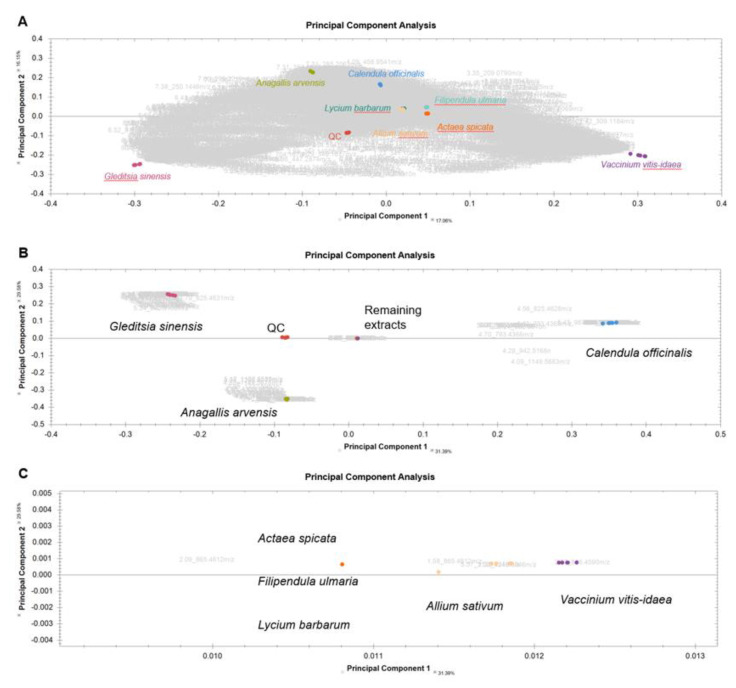
Principal components analysis (PCA) biplot showing scores (colored dots) and loadings (compound IDs in grey with retention time and *m*/*z* values) of UPLC-ESI-IMS-TOF MS runs of selected plant extracts. Different colors refer to different plant species. The QC scores are depicted in red. (**A**) PCA of all compounds (no filters), (**B**) PCA with only possible identifications and (**C**) zoomed-in area.

After identification of some bioactive compounds by GC-MS and the putative identification of several saponins via LC-MS, we tested several commercially available bioactive compounds with our assay. Scopoletin, chlorogenic acid, caffeic acid, quercetin, myricetin, typhaneoside, catechin, gracillin, oleanolic acid and gitogenin (for the last two compounds, only aglycons were commercially available) were applied at concentrations ranging from 10 nM to 10 µM. Similar concentrations have been described in the literature to be relevant for putative antidiabetic properties [42,43,44,45,48]. The results are shown in Figure 6A–T and suggest that only one of the identified bioactive compounds had an influence on the stimulation of insulin secretion when used as a single compound. This single compound, gracillin, showed a strong increase in insulin secretion (2.7-fold) at a concentration of 10 µg/mL but also a significant decrease in cell viability down to 70% at this concentration (see Figure A4).

As a representative insulin secretion suppressing component, only myricetin at a concentration of 10 µM showed an inhibitory effect. At the chosen concentration, insulin secretion decreased by 0.3-fold. None of the other bioactives showed an inhibitory effect.

A large number of plant extracts and natural compounds with insulinotropic effects are currently used for the treatment of diabetes and have already been scientifically explored for their benefits in managing this disease [53]. The constituents and bioactives in these extracts were identified, and it was analyzed whether they play a certain role in insulin secretion. The triterpenoid oleanolic acid, which is widely found in plants, including fruits and vegetables, has several biological effects: oleanolic acid and oleanolic acid glycosides have glucose-lowering effects, which have been demonstrated in vivo, and an insulin secretion-stimulating effect has also been shown in vitro [52,54]. The flavonoid quercetin was identified to stimulate insulin secretion from INS-1 β cells [42]. Additionally, other bioactives, such as scopoletin, which is a type of coumarin, were identified to have antidiabetic properties. Glucose uptake is mediated by insulin, and scopoletin can significantly enhance glucose uptake through the activation of the phosphatidylinositol-3-kinase (PI3K) and adenosine monophosphate-activated protein kinase (AMPK) signaling pathways, resulting in insulin sensitivity improvement [44]. Additionally, myricetin, a natural flavonoid, has been reported to potentiate GSIS in rat islet cells [55]. In our assay, only gracillin showed an insulin secretion stimulatory effect when applied as a single substance, but it also affected cell viability. To determine whether single bioactive compounds modulate insulin secretion, all components of the plant extracts have to be identified. The characterized effects of different plant extracts may also depend on an interaction between different bioactives and not only on the single compounds alone. Further screening and identification of the extracts might reveal additional compounds that could be responsible for the observed effects. Nonetheless, our approach revealed that the main components incorporated functional hydroxyl groups that are present in most bioactive compounds [56].

### 2.5. Impact of Selected Plant Extracts on Mitogen-Activated Protein Kinase Expression

**Figure 7:**
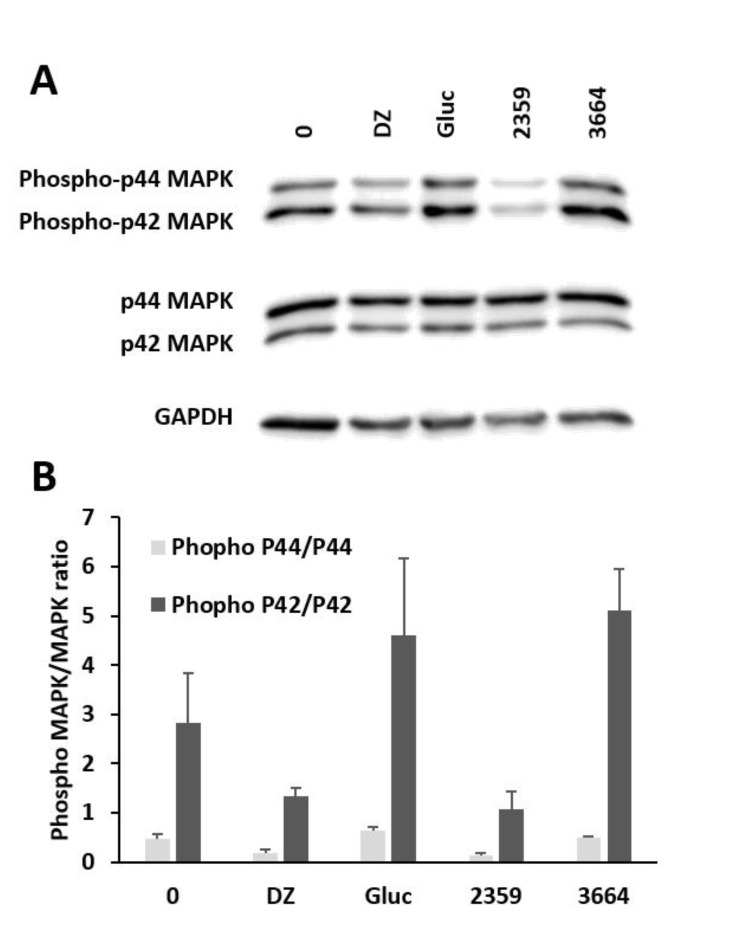
Manipulation of p44/42 phosphorylation. Western blotting (**A**) and quantitative analysis of each band (**B**) of whole-cell extracts from MIN6 β cells after treatment with the indicated substances for 1 h: Blank (0), 250 µM diazoxide (DZ), 10 mM glucose (Gluc), *Filipendula ulmaria* (2359, 10 µg/mL) and *Lycium barbarum* (3664, 10 µg/mL). Mean ± SEM (*n* = 3).

p44/42 Mitogen-activated protein (MAP) kinase is required for insulin secretion from pancreatic β cells. Therefore, we determined the effects of two selected extracts on the expression of this protein. To show the influence of phospho-p44/42 and p44/42, we measured protein levels in MIN6 β cells and demonstrated that these protein levels can be manipulated upon treatment for 1 h with the *F. ulmaria* (2359) and *L. barbarum* (3664) plant extracts, as shown in Figure 7. These effects can be compared to the effects after incubation with glucose or DZ.

Compared to untreated samples, there was an increase in phospho-p42 expression upon treatment with *L. barbarum* extract, which can be compared to treatment with 10 mM glucose, which also showed an increase in protein levels. Our results suggest that glucose and the plant extract of *L. barbarum* induce phosphorylation. DZ was used as a control for substances that inhibit insulin secretion. A decrease in the expression levels of phospho-p42 that was comparable to treatment with the *F. ulmaria* extract was observed. These results suggest that the ERK1/2 signaling cascade also participates in the regulation of the secretion of insulin in living MIN6 β cells.

The p44/42 MAP kinase cascade controls nuclear events in β cells, such as cell differentiation and gene transcription, and ERK1/2 is also required for optimal insulin secretion. It has been described in the literature that blocking the activation of ERK1/2 with different inhibitors results in partial inhibition of GSIS. The ERK1/2 cascade also participates in the phosphorylation of synapsin I, which is associated with the translocation of insulin granules for insulin exocytosis [57]. Our results also suggest that among other kinases, ERK1/2 represents an alternative transduction signal that influences the effects of glucose or certain plant extracts on insulin secretion. It has been reported that the bioactive compound quercetin, which we also found in our plant extracts, potentiates glucose- and glibenclamide-induced insulin release and ERK1/2 phosphorylation [42,58].

ERK1/2 activity is important for optimal insulin secretion and promotion of MIN6 β cell survival. ERK1/2 plays a key role in glucose-mediated pancreatic β cell survival. A disruption in ERK1/2 activity causes impaired protein functions and decreased protein levels of cAMP-responsive element-binding protein (CREB). Performing siRNA knockdown to silence the expression of ERK1/2 proteins also results in high cell mortality [59].

Glucose activates a signaling cascade including the Raf-MEK-ERK MAP kinase pathway, which is activated by PAK1, and MEK1/2—ERK1/2 signaling is important for normal GSIS and F-actin remodeling [60]. In conclusion, the role of ERK1/2 activity in insulin secretion from MIN6 β cells stimulated by glucose, plant extracts or in a basal state needs to be investigated in more detail.

## 3. Materials and Methods

### 3.1. Reagents

Scopoletin, chlorogenic acid, caffeic acid, quercetin, myricetin, catechin, oleanolic acid, gitogenin and gracillin, as well as all solvents and other chemicals were obtained from Sigma-Aldrich Handels GmbH (Vienna, Austria) unless noted otherwise. Coelenterazine (CTZ) was obtained from Carl Roth GmbH (Karlsruhe, Germany). Typhaneoside was purchased from Chemtronica AB (Sollentuna, Sweden). A coelenterazine stock solution was prepared by mixing 1 mg/mL acidified methanol (1.06% HCl in pure methanol) for stabilization, and aliquots were stored at −80 °C. Assay buffer was prepared using phosphate-buffered saline (PBS) supplemented with 0.1% Triton X-100 and 20 mM ascorbic acid as an antioxidant to increase CTZ stability. Ninety-six-well plates were obtained from Greiner Bio-One GmbH (Kremsmünster, Austria). A library containing more than 1500 aqueous plant extracts was provided by PECKISH [32]. The GC-grade derivatization reagent BSTFA (≥99%) with 1% trimethylchlorosilane [TMCS] was obtained from Sigma-Aldrich (Schnelldorf, Germany). GC-MS grade acetonitrile, ethanol, pyridine and toluene were purchased from VWR (AT, Vienna, Austria). Derivatization for GC-MS was performed using a Thermal Shake lite thermoshaker (VWR, Vienna, Austria). An Eppendorf Concentrator 5301 attached to a KNF N 840 Laboport vacuum pump was used for solvent evaporation (Hamburg, Germany).

### 3.2. Cell Culture

Mouse MIN6 β cells stably expressing Ins-GLuc were a kind gift from M. A. Kalwat (UT Southwestern Medical Center, Dallas, TX, USA). For the generation of the luciferase sensor, human insulin with humanized *Gaussia* luciferase was inserted into the C-peptide [20]. Cells were cultured in Dulbecco’s modified Eagle’s medium (DMEM; PAN-Biotech, Aidenbach, Germany) supplemented with 15% fetal bovine serum (FBS), 1% penicillin/streptomycin, 0.5% G418 and 0.1% 2-mercaptoethanol at 37 °C in a humidified atmosphere (≥95%) with 5% CO_2_. For insulin secretion experiments, cells were seeded in 96-well plates at 5 × 10^4^ cells per insert and incubated for 3–4 days.

### 3.3. Cell Viability Assay

Cell viability was evaluated using a resazurin-based in vitro toxicology assay according to the manufacturer’s protocol. Briefly, cells were seeded in 96-well plates at 5 × 10^4^ cells per well, grown to 80% confluence and incubated with the indicated test substances at 37 °C for 2 h. Subsequently, the cells were washed and incubated with 10% resazurin in cell culture medium at 37 °C for 2 h. The level of the reduced form of resazurin (resorufin) was then determined using a microplate reader in fluorescence mode (544 nm excitation, 590 nm emission; POLARstar Omega, BMG LABTECH, Ortenberg, Germany). Data were analyzed using the OmegaMARS Data analysis software package (BMG LABTECH, Ortenberg, Germany). Cell viability was normalized to untreated cells grown under the same conditions. Each test substance was measured at least in quadruplicate.

### 3.4. Insulin Secretion Assay

The insulin secretion assay was adapted from a protocol described by Kalwat et al. [20]. After incubating Ins-GLuc-MIN6 β cells in 96-well plates, the cells were washed twice with 200 µL of Krebs–Ringer-Phosphate-HEPES (KRPH) buffer and starved with KRPH buffer for 1 h at 37 °C. After removing the buffer, the cells were washed again with 200 µL of KRPH buffer before incubation in buffer containing 10 mM glucose, 250 µM DZ or the indicated plant extracts at 37 °C. After 1 h, 50 µL of supernatant was pipetted into a white opaque 96-well plate and mixed with 10 µL of freshly prepared GLuc assay working solution using a multichannel pipette. Next, the CTZ stock solution (1 mg of CTZ in 1 mL of pure methanol supplemented with 1.06% HCl) was mixed at a ratio of 1:100 with assay buffer. Assay buffer was prepared from phosphate buffer supplemented with 20 mM ascorbic acid and 0.1% Triton X-100. GLuc uses CTZ as a single substrate for the implementation of the assay. To test substances that stimulate insulin secretion, luminescence was measured immediately after mixing the supernatant with assay working buffer using a microplate reader in luminescence mode (POLARstar Omega, BMG LABTECH, Ortenberg, Germany). A schematic process overview of insulin secretion stimulation is shown in Figure 1C (1, 2, 3, 4).

To test for plant extracts that suppress insulin secretion, Ins-GLuc-MIN6 β cells were incubated with 10 mM glucose after treatment with different plant extracts for 1 h. Each test substance was measured in quadruplicate. A schematic process overview of insulin secretion suppression is also shown in Figure 1C (1, 2, 5, 6, 7).

### 3.5. Sample and Standard Preparation for GC-MS

Sample cleanup was performed by dilution of each plant extract (20 µL) with 80 µL of acetonitrile. After centrifugation at 17,000× *g*, 80 µL of the supernatant was transferred to fresh screwcap tubes and evaporated to dryness. After the addition of 50 µL of BSTFA and 50 µL of pyridine, derivatization was performed at 80 °C for 60 min at 1100 rpm using a thermoshaker. For GC measurement, 50 µL of the sample was transferred to a glass vial, and 450 µL of toluene was added. Standard substances were dissolved in ethanol and diluted to 10 mg/L. After dilution, the standards were treated in the same manner as the plant extracts. All analytical standards were from Sigma-Aldrich Handels GmbH (Vienna, Austria).

Eight plant extracts were analyzed in selective ion mode (SIM) after derivatization with BSTFA and identification in total ion current (TIC) mode. Compounds were identified using derivatized analytical standards to determine their individual mass spectra and retention times. Derivatization conditions were utilized as suggested by the literature [61].

### 3.6. Instrumentation for GC-MS

Plant extract analysis was performed on a Thermo Trace 1300 GC equipped with a programmable temperature vaporizer (PTV) and a Thermo TSH100 autosampler coupled to a Thermo ISQ 7000 mass spectrometer (Thermo Fisher Scientific, Waltham, MA, USA). Data processing was carried out with Chromeleon 7.2.10 software (Thermo Fisher Scientific, MA, USA).

Chromatographic separation of the plant extracts was achieved using a TRACE TR-5MS (0.25 mm, 0.25 µm, 30 m) column (Thermo Fisher Scientific, Waltham, MA, USA). The PTV injector port temperature was maintained at 90 °C for injection and heated to 300 °C at a rate of 5 °C/s. The GC column temperature was maintained at 90 °C for 2 min, increased from 90 °C to 150 °C at a rate of 10 °C/min, further increased from 150 °C to 320 °C at a rate of 30 °C/min, and then held at 320 °C for 5 min. During the measurements, the transfer line was maintained at 300 °C, and the ion source was maintained at 250 °C. The GC was operated with helium (99.999%) at a constant flow rate of 1.5 mL/min. Each sample was determined via splitless injection of 2.0 µL. The fragment ions at *m*/*z* = 222, 264, 396, 650, 662, 750 and 786 were used in selected ion mode for the identification of caffeic acid, carvacrol, catechin, chlorogenic acid, myricetin, quercetin and scopoletin, respectively. Relative abundancies were determined in TIC mode in the range of *m*/*z* = 50–1000. Ionization was carried out in electron impact (EI) mode at 70 eV.

### 3.7. Western Blot Analysis

Protein expression related to pancreatic β cell metabolism was evaluated using Western blot analysis. MIN6 β cells were seeded in 6-well plates and grown to 80% confluence. After incubation for 2–3 days, the cells were treated with aqueous plant extracts at 10 µg/mL for 2 h and then lysed with Cell Lysis Buffer (Cell Signaling Technology, Frankfurt, Germany) on ice for 5 min. The cell lysates were collected, sonicated and centrifuged at 14,000 rpm for 10 min at 4 °C. The supernatants were collected, and the protein concentration was determined using a Micro BCA Protein Assay Kit (Thermo Fisher Scientific, Waltham, MA, USA). The proteins (20 µg/lane) mixed with 50 mM Tris-HCl buffer (pH 6.8) and 4× sample buffer (200 mM Tris-HCl, 8% SDS, 40% glycerol, 0.4% bromophenol blue, 5% 2-mercaptoethanol) were separated using 10% sodium dodecyl sulfate (SDS) polyacrylamide (PA) gel electrophoresis and transferred to nitrocellulose membranes by semidry transfer (Trans-Blot Turbo Transfer System) (Bio-Rad Laboratories, Hercules, CA, USA). Membranes were blocked for 5 min in EveryBlot Blocking Buffer (Bio-Rad Laboratories, Hercules, CA, USA), followed by incubation with primary antibodies: phospho-p44/42 MAPK (Erk1/2) (Thr202/Tyr204), p44/42 MAPK (Erk1/2), and GAPDH (D16H11) XP^®^ (Cell Signaling Technology, Frankfurt, Germany) for 1 h at room temperature (RT) and thereafter incubated with an anti-rabbit IgG (whole molecule)-peroxidase (Sigma-Aldrich, Vienna, Austria) secondary antibody for 1 h at RT. Specific proteins were detected by the Clarity^TM^ Western ECL Substrate (Bio-Rad Laboratories, Hercules, CA, USA) and visualized by a ChemiDoc^TM^ MP Imaging system (Bio-Rad Laboratories, Hercules, CA, USA). Semiquantitative analysis was performed using Image Lab^TM^ software (Bio-Rad), and the results are presented as an average of three replicas.

### 3.8. Ultra-Performance Liquid Chromatography-Electrospray Ionization-Ion Mobility-Time-of-Flight Mass Spectrometry (UPLC-ESI-IMS-TOF MS)

Plant extracts (10 mg/mL, water) were diluted with water (1:5), sonicated (5 min), membrane-filtered (0.45 µm), and analyzed in five replicates (3 µL) by means of UPLC-ESI-MS-TOF MS on a Waters Vion HDMS mass spectrometer (Waters, Manchester, UK) coupled to an ACQUITY I-Class UPLC system (Waters, Milford, MA, USA) equipped with a 2.1 × 150 mm, 1.7 µm BEH C18 column (Waters, Milford, MA, USA) consisting of a binary solvent manager, sample manager, and column oven. Using a flow rate of 0.4 mL/min at 45 °C, the following gradient was used for chromatography: starting with a mixture (5/95, *v*/*v*) of aqueous formic acid (0.1% in H_2_O) and ACN (0.1% formic acid), the ACN content was increased to 100% within 8 min, kept constant for 1 min, decreased to 5% within 0.4 min, and finally kept constant for 0.6 min at 5%. The scan time for the HDMSE method was set to 1.0 s. Analyses were performed in negative ESI sensitivity mode using the following ion source parameters: capillary voltage 2.3 kV, source temperature: 120 °C, desolvation temperature: 450 °C, cone gas flow: 50 L/h, and desolvation gas flow: 850 L/h. Data were processed using UNIFI 1.8 (Waters, Milford, MA, USA). All data were lock-mass corrected on the pentapeptide leucine enkephalin (Tyr-Gly-Gly-Phe-Leu, *m*/*z* 554.2615 [M-H]-) in a solution (100 pg/mL) of ACN/0.1% formic acid (1/1, *v*/*v*). The scan time for the lock mass was set to 0.2 s with an interval of 0.5 min. Calibration of the MS in the range from *m*/*z* 50 to 1200 was performed using a solution of MajorMixTM (Waters). UPLC-MS was performed with UNIFITM software (Waters, Milford, MA, USA). The collision energy ramp for HDMSE was set from 20 to 60 eV. Further details of the Vion IMS QToF instrument and processing and detection parameters were adapted from [62].

For quality control (QC reference), a pooled sample of all 8 plant extracts was used for automatic normalization in Progenesis QI (vs. 4.0) software (Waters, Milford, MA, USA) and error correction of the detected MS signals.

The raw data obtained from UPLC-ESI-IMS-TOF MS analysis were processed with Progenesis QI using the following peak picking conditions: all runs, automatic limits, sensitivity 3, and no retention time limits. In total, 45 profile MSE raw data were imported and processed automatically. Tag filtration was carried out by means of ANOVA *p*-value ≤ 0.05 and maximum fold change ≥ 2 to identify significant compound differences between the groups. To identify possible saponins, an in silico fragment database was created by defining the MetaScope search parameters using an automatic detection format with a precursor tolerance of 5 ppm and a fragment tolerance of 5 ppm. The database contained structural information on a total of 56 saponins [51,52,54,63,64,65,66] known from the literature from the individual plant extracts. The list of compounds was compared to this database. Compounds used for principal component analysis (PCA) were filtered by possible hits of this database.

### 3.9. Statistical Analysis

The results are expressed as the mean ± standard error of the mean (SEM) unless stated otherwise. Statistical significance was determined by Student’s *t*-test. Values of *p* less than 0.05 were considered statistically significant. For plant extract screening, the z-score was calculated using the formula z = (x − µ)/σ, where x is the median of the sample data, µ is the median of all plant extracts and σ is the standard deviation of all plant extracts. For statistical analysis of the UPLC-ESI-IMS-TOF MS data, Progenesis QI (vs. 4.0) (nonlinear Dynamics, Waters, Milford, MA, USA) was used.

## 4. Conclusions

Using a high-content screen, we identified several plant extracts from a library of 1100 samples that stimulate or inhibit insulin secretion in living MIN6 β cells. Some of them might be of interest for application in pharmaceuticals or nutraceuticals. Chemical analysis of the most promising candidates resulted in the identification of numerous bioactive compounds. Two bioactive compounds with insulin secretion modulating properties were identified: on the one hand, myricetin shows an insulin secretion inhibiting effect at a concentration of 10 µM, and on the other hand gracillin, a stimulating compound. Due to the toxic effects of gracillin, a definite giving of evidence concerning the insulin secretion properties cannot be made. However, most of the tested compounds could not be linked to the observed biological activity as a pure compound. It is likely that not only a single bioactive compound but also the synergistic action of several bioactives in certain ratios contributes to the insulin secretion modulating effect. Another reason could be that the adequate compound was not tested; therefore, bioactives responsible for insulin secretion modulating activity remained undiscovered. Furthermore, we depicted the dose-dependent effects of various insulinotropic plant extracts on the viability of MIN6 β cells.

## Data Availability

The data presented in this study are available in the main text and in the Appendix A.
